# Multi-organ spatial stratification of 3-D dose distributions improves risk prediction of long-term self-reported severe symptoms in oropharyngeal cancer patients receiving radiotherapy: development of a pre-treatment decision support tool

**DOI:** 10.3389/fonc.2023.1210087

**Published:** 2023-08-08

**Authors:** Andrew Wentzel, Abdallah S. R. Mohamed, Mohamed A. Naser, Lisanne V. van Dijk, Katherine Hutcheson, Amy M. Moreno, Clifton D. Fuller, Guadalupe Canahuate, G. Elisabeta Marai

**Affiliations:** ^1^ Department of Computer Science, The University of Illinois Chicago, Chicago, IL, United States; ^2^ Department of Radiation Oncology, The University of Texas MD Anderson Cancer Center, Houston, TX, United States; ^3^ Department of Electrical and Computer Engineering, University of Iowa, Iowa City, IA, United States

**Keywords:** radiation therapy, clustering, head and neck cancer, stratification, symptom burden, quality of life

## Abstract

**Purpose:**

Identify Oropharyngeal cancer (OPC) patients at high-risk of developing long-term severe radiation-associated symptoms using dose volume histograms for organs-at-risk, via unsupervised clustering.

**Material and methods:**

All patients were treated using radiation therapy for OPC. Dose-volume histograms of organs-at-risk were extracted from patients’ treatment plans. Symptom ratings were collected via the MD Anderson Symptom Inventory (MDASI) given weekly during, and 6 months post-treatment. Drymouth, trouble swallowing, mucus, and vocal dysfunction were selected for analysis in this study. Patient stratifications were obtained by applying Bayesian Mixture Models with three components to patient’s dose histograms for relevant organs. The clusters with the highest total mean doses were translated into dose thresholds using rule mining. Patient stratifications were compared against Tumor staging information using multivariate likelihood ratio tests. Model performance for prediction of moderate/severe symptoms at 6 months was compared against normal tissue complication probability (NTCP) models using cross-validation.

**Results:**

A total of 349 patients were included for long-term symptom prediction. High-risk clusters were significantly correlated with outcomes for severe late drymouth (p <.0001, OR = 2.94), swallow (p = .002, OR = 5.13), mucus (p = .001, OR = 3.18), and voice (p = .009, OR = 8.99). Simplified clusters were also correlated with late severe symptoms for drymouth (p <.001, OR = 2.77), swallow (p = .01, OR = 3.63), mucus (p = .01, OR = 2.37), and voice (p <.001, OR = 19.75). Proposed cluster stratifications show better performance than NTCP models for severe drymouth (AUC.598 vs.559, MCC.143 vs.062), swallow (AUC.631 vs.561, MCC.20 vs -.030), mucus (AUC.596 vs.492, MCC.164 vs -.041), and voice (AUC.681 vs.555, MCC.181 vs -.019). Simplified dose thresholds also show better performance than baseline models for predicting late severe ratings for all symptoms.

**Conclusion:**

Our results show that leveraging the 3-D dose histograms from radiation therapy plan improves stratification of patients according to their risk of experiencing long-term severe radiation associated symptoms, beyond existing NTPC models. Our rule-based method can approximate our stratifications with minimal loss of accuracy and can proactively identify risk factors for radiation-associated toxicity.

## 1 Introduction

With advancements in precision radiation therapy and the emerging dominance of HPV-driven tumors over smoking-related tumors (1), patient survival has improved significantly for Oropharyngeal Cancer (OPC) patients (2, 3). Despite this, survivors that receive radiation therapy frequently suffer severe lasting side effects that can significantly reduce quality of life following treatment as a side effect of radiation-induced damage to organs, such as xerostomia (drymouth) or difficulty swallowing (4). Damage to vital organs such as salivary glands and swallowing muscles from radiation is a major factor in reduced quality of life, and precisely determining the risk associated with patient treatment plans can help physicians improve patient endpoints in two ways (5). First, it allows oncologists to identify which organs to prioritize when designing individualized treatment plans. Second, when risk of organ damage is unavoidable, oncologists can prescribe preventative treatments, such as occupational therapy, to minimize side effects.

Existing approaches to radiation treatment planning often consider single-value dose thresholds for key organs (6). For xerostomia, existing guidelines recommends limiting the mean dose to the parotid glands to under 20Gy to the contralateral side, or 25Gy for the ipsilateral side (7), although other research suggests higher dose thresholds of 35.7Gy (8). Single-dose thresholds are useful in their practicality for clinical researchers but suffer from poor generalizability and fail to consider interactions between multiple organs, or effects from different dose distributions that yield similar mean doses.

Other approaches such as Normal Tissue Complication Probability (NTCP) models attempt to account for 3-dimensional dose distributions to organs by considering the contribution for different parts of the dose-volume histogram to output a final risk probability (9). Existing xerostomia NTCP models mainly consider mean doses to organs at risk (10). NTCP models can outperform dose thresholds but suffer from higher complexity that may lead to overfitting on the data, and are difficult to use for dose planning (11). More complex deep learning models have shown good performance in predicting patient endpoints (12). However, research has suggested that despite improvements in performance from deep learning models, they don’t outperform standard statistical approaches in practice due to their poor transparency and generalizability (13).

To address this problem, we present an unsupervised learning method for stratifying patients based on 3D dose distributions to relevant organs-at-risk, to identify clusters of patients that are at risk of radiation-associated long-term severe symptoms after treatment. By using clusters as proxies for risk, these clusters can serve as risk stratifications for patient symptoms that account for complex dose distributions to multiple organs at risk, while maintaining simplicity and actionability not seen in NTCP or more complicated models. To translate these stratifications into more actionable doses, we also propose a method of producing a set of dose thresholds to approximate the high-risk group. Focusing on predicting patient-reported drymouth, we compare our risk stratification to existing dose-based models and models using clinical factors to show that our cluster-based and simplified threshold-based stratifications can be used to improve risk predictions of self-reported symptoms.

## 2 Methods

### 2.1 Overview

**Figure 1:**
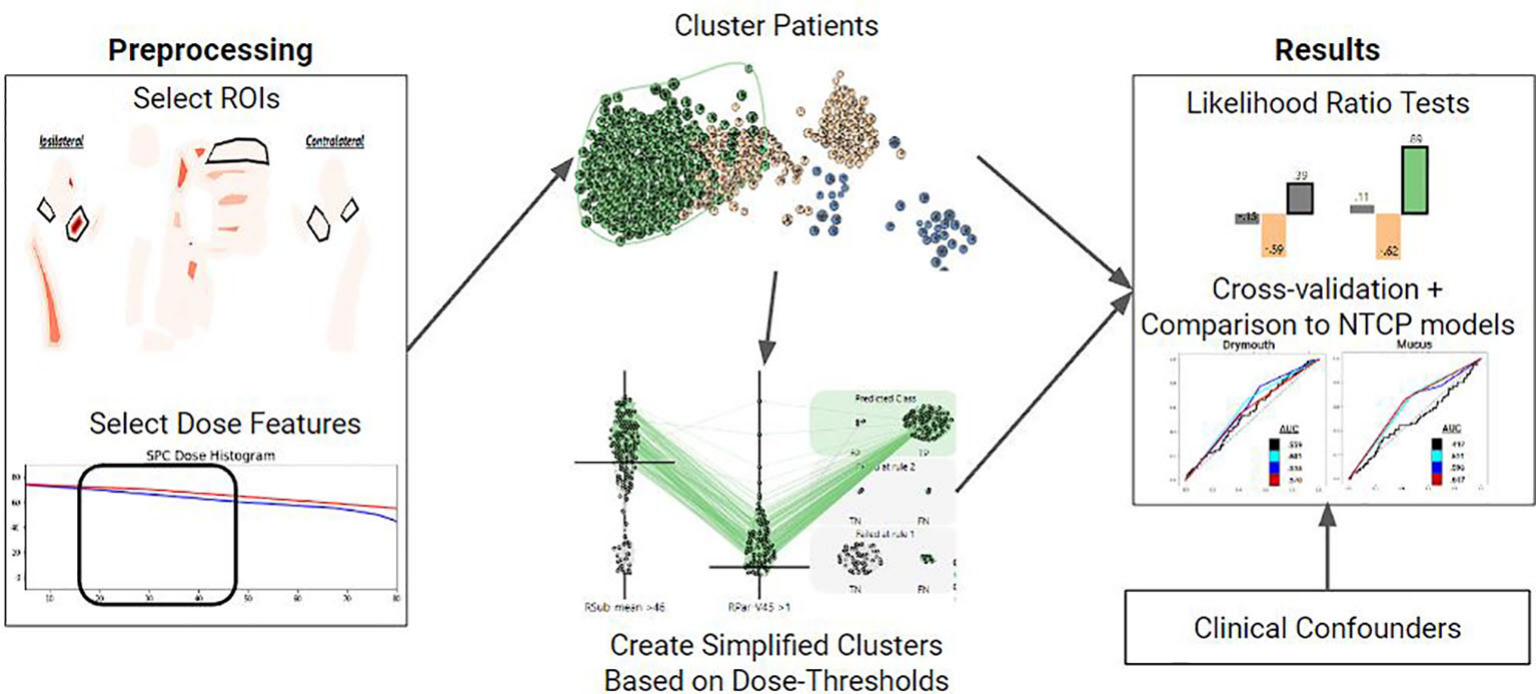
Overview of the methods used for each symptom of interest. First, relevant ROIS and DVH features are selected. These features are used to vectorize each patient and cluster them using a Gaussian Mixture Model. Clusters are then converted into a set of dose thresholds to approximate the high-risk group. Both clusters and simple clusters are evaluated using multivariate likelihood-ratio tests and cross-validation against NTCP models with clinical covariates to assess how predictive they are of the symptom of interest at 6 months.

We detail our methods in the following sections as follows: (1) diagnostic and treatment data is collected and preprocessed from a cohort of Oropharyngeal cancer patients. We then filter out relevant patients and preprocess relevant features. (2) Patient treatment plans are fed into a clustering algorithm in order to extract patient risk clusters. (3) Ruling mining is used to produce a set of dose thresholds that approximate the high-risk cluster. (4) We perform multivariate correlation testing to show that the clusters are correlated with severe long-term toxicities. (5) We perform cross-validation using logistic regression to compare the performance of our clusters to normal-tissue complication probability models. An overview of our process is shown in (**Figure 1**). The remainder of this subsection details an overview of our methodology.

First, we select a set of dosimetric features for organs relevant to each toxicity, and cluster patients based on these features into three clusters that correspond to low, medium, and high dose groups. We then identify the high-dose group, which is assumed to be the group at higher risk of long term drymouth due to damage to the relevant organs. Thus, inclusion in this high dose cluster can be used as a stratification metric for risk of tissue damage. In order to produce a more actionable and explainable stratification, we also identify a minimal set of dose thresholds to organs at risk which closely models membership in this high-risk group.

For this paper, we consider the following four self-reported symptoms: drymouth, difficulty swallowing (swallow), excessive mucus (mucus), and voice dysfunction (voice). Drymouth has been shown to be an accurate indication of salivary function (14), and other symptoms are included as we theorize that they are also causally linked to damage to key tissues. Separate feature sets (choice of organ and dose thresholds) and clusters are generated for each symptom.

Our symptom data is self-reported ratings of symptoms at their worst between 0 (none) and 10 (the worst I can imagine) taken from the MD Anderson Symptom Inventory (15). To identify long-term outcomes, we consider the reported symptom rating during the patient’s 6 month (late) followup. We consider whether the reported symptom is > 4 (severe), as well as the change in reported symptom from the patient’s reported drymouth at the start of treatment is > 4 (severe change). These result in 2 binary outcomes for each symptom. Values measured during treatment were only used for imputing baseline values.

We demonstrate that our stratifications are highly correlated with self-reported late symptoms using multivariate likelihood ratio tests, and well as cross-validation to demonstrate that the clusters provide better predictive performance for late symptoms relative to existing clinical and normal tissue complication probability models (7, 10, 16), while being more explainable and accessible in real settings.

### 2.2 Data collection and preprocessing

Data were collected retrospectively from a continuously enrolled cohort of Oropharyngeal patients treated using curative-intent Radiation Therapy at the MD Anderson Cancer Center between 2010 and 2021. DVH histograms were collected from pre-treatment CECT scans taken prior to the start of treatment. Organs of interest were segmented, and dose-volume histograms were extracted using commercially available software (17), as described in (18). Additional information such as T-stage, N-stage (19), HPV/p16 status, tumor location, demographic information, and initial ECOG performance score (20) was collected from electronic health record data. T and N stage are existing risk stratifications based on the size and spread of primary and secondary tumors, respectively, while ECOG performance score is an indicator of the patient’s level of functioning at the start of treatment.

To collect symptom information, patients were asked to fill out an MD Anderson symptom inventory (MDASI) questionnaire (15) at weekly intervals during treatment, as well as during follow up sessions at 6 weeks, and 6 months after treatment, for a maximum of 9 time points. These questionnaires asked patients to rate the severity of 28 side effects, including drymouth, on a scale of 0-10.

Inclusion criteria for the patients were: 1) presence of OPC confirmed via biopsy; 2) patient was treated using curative-intent IMRT; 3) dose-volume histogram data available for organs at-risk in the head and neck; 4) at least 70% of the items on the MDASI questionnaire are available in the time period from the start of treatment until 6 months after treatment; 5) symptom ratings available at 6 months; 6) patients survived long enough for a 6 month follow up appointment. The final cohort consisted of 349 patients.

Because baseline ratings were not available for 59 (16.9%) patients, we used a denoising neural network (21) to impute missing values from related symptoms and the ratings at other time points for patients with enough symptom ratings. To train the symptom imputation model, all symptom ratings from all 10 time points were used as input data. To ensure that enough symptom data was available to impute missing values, we only considered patients with a baseline drymouth rating and with at least 70% of all symptom ratings across all timesteps available. In order to train the network to learn to impute missing data, we used gaussian dropout during training, where values were randomly get to 0 with a 50% change during training, and the network was trained to reconstruct the original values using the other symptom ratings. The denoiser used two fully connected layers with a ReLU activation function followed by batch normalization. The model was trained using the Adam optimizer and mean-squared-error loss with a learning rate of.001 for 2000 with early stopping. The final model had a mean reconstruction error of 6.18%

### 2.3 Clustering

In order to demonstrate that our approach can be generalized to any outcome that is associated with radiation-induced tissue damage, we apply our methodology for identifying high-risk clusters for predicting late severe ratings for four different symptoms: drymouth, swallow, mucus, and voice. Optimal cluster parameters were identified using a previously published visual analytics system developed for this project (22). For all outcomes, we use 3 clusters, and consider the cluster with the highest total mean dose to organs at risk to be the “high-dose cluster”. Organs and DVH values used for each symptom cluster are given in (**Table 1**). To account for bilaterality of the head, we consider the side with the higher total mean dose as the primary side and encode the parotid and submandibular glands on that side as the “ipsilateral side”, and the organs on the other side as the “contralateral” side.

**Table 1:** Table of rules used to approximate the high-dose clusters for alternative outcomes, along with the precision, recall, and info gain associated with each set of simplified clusters, to show how well the simplified clusters approximate the high-dose group.

Outcome	Cluster Organs	Cluster DVH Features	Thresholds	N (HD)	N (Simplified HD)	Cluster Precision	Cluster Recall	NTCP Organs
Drymouth	Both Parotid Glands, Both Submandibular Glands, Hard Palate	V25-V60	Contralateral submandibular gland V45 > 61	219	205	0.98	0.89	Parotid glands, Submandibular glands, soft palate, upper lip, lower lip, oral cavity, mylogeniohyoid
			Contralateral parotid gland V45 > 0					
Swallow	IPC, MPC, Supraglottic Larynx, Esophagus, Mylogeniohyoid Muscle	V30-V65	IPC V50 > 40	60	65	0.892	0.967	IPC, SPC, Supraglottic Larynx, Parotid Gland, Cricopharyngeal Muscle
			Supraglottic Larynx V60 > 46					
Mucus	Both Parotid Glands, Both Submandibular Glands	V25-V65	Contralateral Submandibular Gland V50 > 48	184	171	0.988	0.918	Soft Palate, Hard Palate, Oral Cavity, Mandible, Tonge, Parotid Glands
			Contralateral Parotid Glannd V45 > 0					
Voice	Tongue, IPC, Larynx, Supraglottic Larynx, Contralateral Submandibular Gland	V45-V65	IPC V55 > 34	45	5.50E+01	8.00E-01	0.978	Larynx, Supraglottic Larynx, Tongue, Genioglossus Muscle, Mylogeniohyoid Muscle
			Larynx Max Dose > 66					

For example, when creating clusters for drymouth, we used the doses to both parotid glands, both submandibular glands, and the hard palate. We then considered the following DVH features from each organ of interest: The dose delivered to 25% of the volume (V25) through the dose delivered to 60% of the volume (V60), collected in increments of 5%, which were selected by identifying the dose features with the maximum mutual information with all late patient symptoms. Each patient was thus encoded as a vector of 40 (5 organs x 8 features) values.

The patient dose distribution was modeled using a Bayesian Gaussian Mixture Model (BGMM), an unsupervised machine learning model that learns from the distribution of the data (23). We chose to use mixture models as we found that they proved to be effective at modeling patterns in the dose distribution due to difference in the position of the underlying tumors (24). We consider the bayesian variant of the model as it is traditionally less sensitive to the choice of parameters (25). After training a three cluster BGMM, the patients were clustered by assigning them to the component with the maximum likelihood.

### 2.4 Simplified cluster generation

In this paper we are mainly interested in high-dose, high-risk patients. To define the high dose (HD) group as follows. First, we calculate the mean dose for the organs of interest used to define the clusters. We then calculate the sum of the mean doses for each cluster and consider the cluster with the highest total mean dose to be the HD group. We verify that this HD group is also the group with the highest incidence of severe late symptom ratings.

To make the model more accessible for users without access to the original model, we also generate a “simplified” high risk group (SHD) as follows. First, we look at all dose features for all organs used in the cluster (e.g., V55 to the parotid gland). For each feature, we test different value thresholds to split the cohort into 2 groups (e.g., V55 to the parotid > 1). We then calculate the mutual information between this split, and the HD cluster, and select the 25 feature splits with the highest mutual information gain. For each rule, we then repeat this process only on the sub-cohort that meets the criteria of the first rule and select the 25 sets of 1-2 feature splits with the highest mutual information gain. We repeat this process iteratively until we identify a set of dose thresholds that maximize the mutual information with cluster membership. The group that exceeds all thresholds in the data is considered the “simplified” high dose (SHD) group. This results in a set of rules that can quickly approximate the original HD group, while providing thresholds that may be used for soft constraints when planning treatment plans.

Once the high-risk and simplified high-risk clusters were identified, we performed a chi2 test between clinical covariate and membership in either the original clusters or the simplified cluster. T-test statistic and significance levels were collected for the following covariates: Sex (male/female), T-stage, N-stage, HPV p16 status, primary tumor subsite, radiation treatment type, if the patient had surgery prior to treatment, age, total dose to the primary tumor, and the dose-fraction.

### 2.5 LRT tests

For each endpoint we assess the predictive power of the original and simplified clusters using a likelihood ratio test (LRT). For this, we build maximum likelihood estimation models that consider clinical covariates as well as models that include both clinical covariates and either all clusters or each cluster individually. We then perform an LRT to identify if the goodness of fit of the model with clusters added has a statistically significant better fit than the baseline cluster with only clinical covariates. Additionally, we consider the linear case where we model the outcome on a 10-point scale using linear regression. We report the p-values from the likelihood ratio test, the odds ratios are taken from the model coefficients for each cluster, and the change in Akaike (AIC) and Bayesian (BIC) information criteria between each model and the clinical baseline mode. AIC and BIC are estimates of the goodness of fit of a model that includes a penalty for the number of variables considered, in order to prevent overfitting, where lower scores indicate better fits (26). For BIC, reductions in score relative to the baseline model of at least 2 indicate reasonable evidence, while reductions of at least 6 indicate “strong” evidence of improvement (27).

For the purpose of testing our models, we consider the following covariates that serve as our clinical confounders: T-stage > 2 (T-stage); N-stage > 1 (Nn-stage); HPV/p16 status (hpv); primary tumor at the base of the tongue (BOT); primary tumor at the Tonsil (Tonsil); age >= 65 years at the time of diagnosis (age); ECOG performance score = 1; ECOG performance score = 2 (ECOG score); and if the patient had a mean dose of > 20 Gy to both parotid glands, or > 25 Gy to one parotid gland (Parotid Limit). These encodings were chosen as they are clinically relevant confounders that have been found to be most relevant when considering treatment type and outcomes. Sex was not included as it was found to not have any correlation with any outcome (p >.8) via chi-squared test, and 90% of the cohort was male. We chose to include T-stage, N-stage, and HPV status separately as our earlier work suggested that T-stage was more predictive of dysphagia than AJCC status (28), which was designed to be predictive of survival, and our cohort had a combination of AJCC 8th edition and 7th edition ratings.

To understand how our baseline confounders compare to our clusters, we performed multivariate maximum likelihood estimation to determine the odds ratio and p-value from the likelihood ratio test between each confounder and outcome individually. Additionally, we tested the correlation between published dose thresholds to organs in the head and neck and severe late drymouth. We also looked at correlations with published dose limits to organs of interest. Rules for dose limits are described in (**Table 2**).

**Table 2:** Description of the dose limits considered to different organs (7), and the toxicity they are designed to avoid.

Organ	Dose Limit (Gy)	Outcome
Spinal Cord	Max dose > 50	Myelopathy
Parotid Gland	Mean dose > 25 for one OR Mean dose > 20 for both	Xerostomia
Inferior Pharyngeal Constrictor (IPC)	Mean dose > 50	Feeding Tube
Inferior Pharyngeal Constrictor (IPC 2)	Mean dose > 60	Aspiration
Medial Pharyngeal Constrictor (MPC)	Mean dose > 50	Feeding Tube
Medial Pharyngeal Constrictor (MPC 2)	Mean dose > 60	Aspiration
Superior Pharyngeal Constrictor (SPC)	Mean dose > 50	Feeding Tube
Superior Pharyngeal Constrictor (SPC 2)	Mean dose > 60	Aspiration
Mandible	Max dose > 70	Osteoradionecrosis
Larynx	V50 > 27	Edema
Brachial Plexus	Max dose > 60	Nerve Damage
Esophagus	V35 > 50 OR V50 > 40 OR V70 > 20 OR V60 > 30	Esophagitis

### 2.6 Cross-validation

In order to compare our model to existing models, we compare cross-validation performance of our clusters (3-level stratification) to a baseline NTCP model based on previous literature. For the NTCP model, we use logistic regression with clinical covariates as well as the dosimetric values to organs at risk that best approximated existing clinical models based on available segmentation data (10, 16). For each outcome, we re-calibrate the NTCP model on the training data during cross-validation in order to ensure the optimal performance of the NTCP model for comparison. All dosimetric values for NTCP models consider the mean dose to the organs considered. For example, the final dose values considered in the NTCP model are the mean doses to the following organs: parotid glands, submandibular glands, soft palate, upper lip, lower lip, oral cavity, and mylogeniohyoid muscle. We included the mylogeniohyoid muscle as we did not have separate contour data for sublingual salivary glands.

When evaluating the performance of our clusters during cross-validation, we rank each cluster based on the number of patients that experience the given outcome in the training data and assign risk to patients in the test data based on the rank of their clusters. In this way, the highest-risk cluster is given a risk score of 1, while the second highest-risk cluster is given a risk score of.5. For the simplified cluster, we always assign a risk of 1 to the high-dose cluster and 0 otherwise. For the whole dataset, this is the equivalent of using the clusters as a xerostomia risk stratification.

We report the area under the receiver-operator curve (AUC-ROC score), which is a measure of the specificity of a test as the sensitivity threshold changes (29); and the Mathew’s correlation coefficient (MCC) (30), which is a special case of a correlation coefficient that has been shown to be useful for evaluating binary outcomes for imbalance data (31), of our risk stratification compared to the baseline and NTCP models for all binary outcomes.

## 3 Results

### 3.1 Demographics

The distribution of patient symptom ratings is shown in (**Table 3**). We see drymouth is the most prevalent symptom, with late severe drymouth occurring in 43.8% of patients and an average rating of 4.34 at 6 months, followed by severe mucus, which only occurs in 16% of patients (mean rating 2.26). Voice had the lowest number of patients with an average rating of 1.07 and only 4% of patients reporting severe voice dysfunction and only 1.7% reporting an increase of at least 5 point from baseline at 6 months.

**Table 3:** Distribution of each symptom rating at 6 months, as well as the number of patients who have ratings or change in ratings above different thresholds, corresponding to “any”, “moderate”, and “severe”.

Symptom	Avg Rating	Rating 5% CI	Rating Median	Rating 95% CI	Threshold	Above Threshold	Above Threshold (%)	Change Above Threshold	Change Above Threshold (%)
Drymouth	4.34	0.4	4	9	0	331	94.8%	295	84.5%
					2	241	69.1%	203	58.2%
					4	153	43.8%	114	32.7%
Swallow	2.14	0	2	7	0	259	74.2%	199	57.0%
					2	112	32.1%	73	20.9%
					4	46	13.2%	29	8.3%
Mucus	2.26	0	2	8	0	255	73.1%	202	57.9%
					2	120	34.4%	86	24.6%
					4	56	16.0%	42	12.0%
Voice	1.07	0	0	4	0	167	47.9%	133	38.1%
					2	51	14.6%	34	9.7%
					4	14	4.0%	6	1.7%

Demographics and demographics within the high-dose and simplified high-dose clusters for each outcome are shown in (**Table 4**). The cohort was predominantly male (90%) and HPV/p16 positive (81%), with a mean age of 59 (95% CI 58-60). A majority of patients were treated with volume-modulated arc therapy or intensity modulated proton therapy (63%), while only 2 patients received 3d conformational therapy. 10% of patients underwent surgery prior to radiation therapy.

**Table 4:** Patient demographics, treatment information of the cohort, as well as the distribution of features within the high-dose (HD) and simplified high-dose (SHD) clusters for each outcome.

Feature		Cohort	Drymouth		Swallow		Mucus		Voice	
	Value		HD	SHD	HD	SHD	HD	SHD	HD	SHD
	Total	349	193	175	60	65	184	171	45	55
Sex	Male	314 (90%)	171 (89%)	155 (89%)	58 (97%)	63 (97%)	163 (89%)	152 (89%)	43 (96%)	53 (96%)
T-stage	T3	48 (14%)	31 (16%)	24 (14%)	13 (22%)	15 (23%)	28 (15%)	22 (13%)	11 (24%)	13 (24%)
	T4	34 (10%)	32 (17%)	31 (18%)	16 (27%)	15 (23%)	31 (17%)	31 (18%)	14 (31%)	15 (27%)
N-stage	N2a/N2b	164 (47%)	88 (46%)	81 (46%)	31 (52%)	36 (55%)	85 (46%)	81 (47%)	21 (47%)	28 (51%)
	N2C/N3	48 (14%)	40 (21%)	40 (23%)	13 (22%)	12 (18%)	40 (22%)	40 (23%)	10 (22%)	13 (24%)
HPV	Unknown	43 (12%)	23 (12%)	20 (11%)	3 (5%)	4 (6%)	22 (12%)	19 (11%)	3 (7%)	3 (5%)
	HPV +	282 (81%)	155 (80%)	142 (81%)	47 (78%)	52 (80%)	147 (80%)	139 (81%)	35 (78%)	42 (76%)
Subsite	BOT	162 (46%)	117 (61%)	114 (65%)	45 (75%)	49 (75%)	113 (61%)	112 (65%)	32 (71%)	40 (73%)
	Tonsil	145 (42%)	52 (27%)	40 (23%)	11 (18%)	13 (20%)	48 (26%)	38 (22%)	10 (22%)	11 (20%)
Treatment	3D Conf.	2 (1%)	0 (0%)	0 (0%)	0 (0%)	0 (0%)	0 (0%)	0 (0%)	0 (0%)	0 (0%)
	VMAT/IMPT	221 (63%)	134 (69%)	120 (69%)	50 (83%)	52 (80%)	128 (70%)	118 (69%)	39 (87%)	46 (84%)
	IMRT	74 (21%)	41 (21%)	39 (22%)	6 (10%)	8 (12%)	39 (21%)	38 (22%)	2 (4%)	5 (9%)
Prior Surgery	Yes	36 (10%)	13 (7%)	13 (7%)	1 (2%)	2 (3%)	12 (7%)	13 (8%)	0 (0%)	0 (0%)
ECOG Perf. Score	1	64 (18%)	41 (21%)	35 (20%)	13 (22%)	17 (26%)	37 (20%)	34 (20%)	12 (27%)	13 (24%)
	2	6 (2%)	3 (2%)	0 (0%)	1 (2%)	1 (2%)	1 (1%)	0 (0%)	0 (0%)	0 (0%)
	Unknown	18 (5%)	8 (4%)	8 (5%)	3 (5%)	3 (5%)	9 (5%)	8 (5%)	3 (7%)	3 (5%)
Age	Mean (95% CI)	59 (58 - 60)	59 (58 - 61)	60 (58 - 61)	63 (61 - 66)	64 (61 - 66)	59 (58 - 61)	60 (58 - 61)	64 (61 - 67)	63 (61 - 65)
RT Dose	Mean (95% CI)	53 (51 - 55)	51 (47 - 55)	51 (48 - 55)	51 (43 - 57)	52 (46 - 58)	52 (49 - 55)	51 (47 - 55)	53 (46 - 60)	54 (47 - 61)
Dose- Fraction	Mean (95% CI)	26 (25 - 28)	26 (23 - 28)	25 (23 - 26)	24 (21 - 27)	25 (22 - 28)	26 (24 - 28)	24 (23 - 26)	25 (21 - 29)	26 (22 - 28)

Continuous values show mean values and 95% confidence intervals within each group. Legend) T-stage: AJCC 8th edition T-staging; N-stage: AJCC 8th edition N-staging; HPV) Whether the patient was HPV/p16+; Subsite: site of primary tumor (BOT, Tonsil, other); BOT, Base of Tongue; VMAT, volumetric modulated arc therapy; IMPT, intensity modulated proton therapy; IMRT, intensity modulated proton therapy; ECOG Perf. Score, Eastern Cooperative Oncology Group pre-treatment performance score; RT Dose: total prescribed RT dose the the main tumor; Dose-fraction: weekly dose delivered to the main tumor.

**Figure 2:**
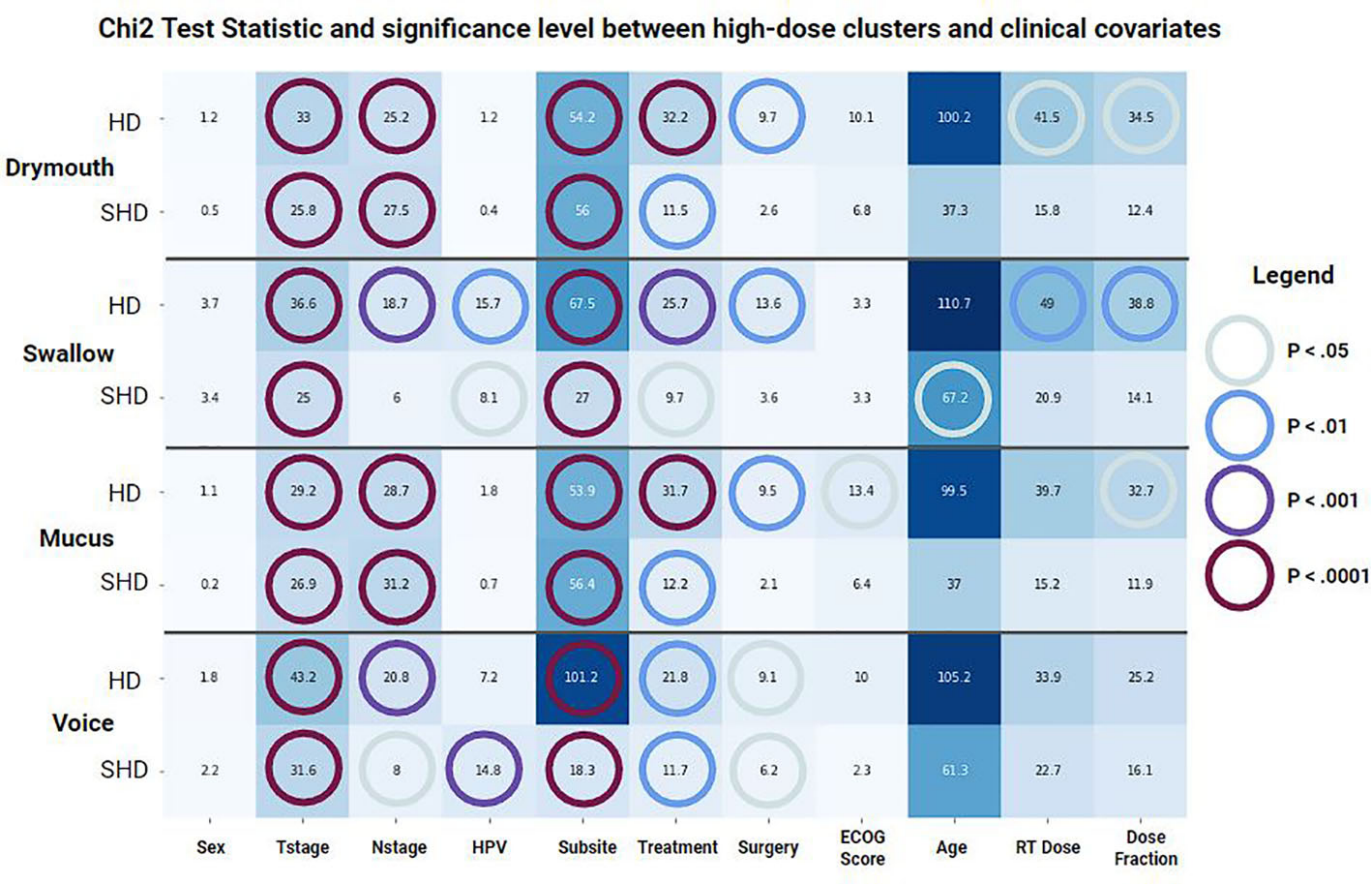
Results of a chi-squared test between covariates and membership in each set of clusters for each outcome. (HD) Standard clusters, (SHD) Simplified clusters. Color and annotations encode t-statistic values while colored circles represent the significance level based on the p-value.

Results of chi-squared tests between demographic features and cluster membership is shown in (**Figure 2**). A significant correlation was found between all cluster memberships and T-stage (p <.0001), tumor subsite (p <.0001), and treatment modality (p <.05), while N-stage was correlated with all but simplified swallowing risk (p <.05). Patients in high-risk clusters had higher rates of stage T4 (10% vs 17-31%) and N2C/N3 tumors (14% vs 18-23%), which correspond to patients likely to receive the most aggressive treatment. Additionally, all high-risk groups had higher incidences of tumors at the base of the tongue (BOT), and lower incidence of tumors in the Tonsil. There was also a higher rate of patients that received VMAT/IMPT in the high-risk clusters (63 vs 69-87%). All standard clusters as well as simplified voice clusters were correlated with lower rates of pre-treatment surgery (p <.05, 10% vs 0-7%). No significant difference was found between ECOG performance score and clusters. Drymouth and Mucus clusters were not correlated with HPV status (p >.05), but there were fewer HPV+ patients in the swallow high-dose (81% vs 78%, p <.01) and simplified high dose clusters 81% vs 80%, (p <.05), as well as simplified voice (81% vs 76%, p <.001).

**Figure 3:**
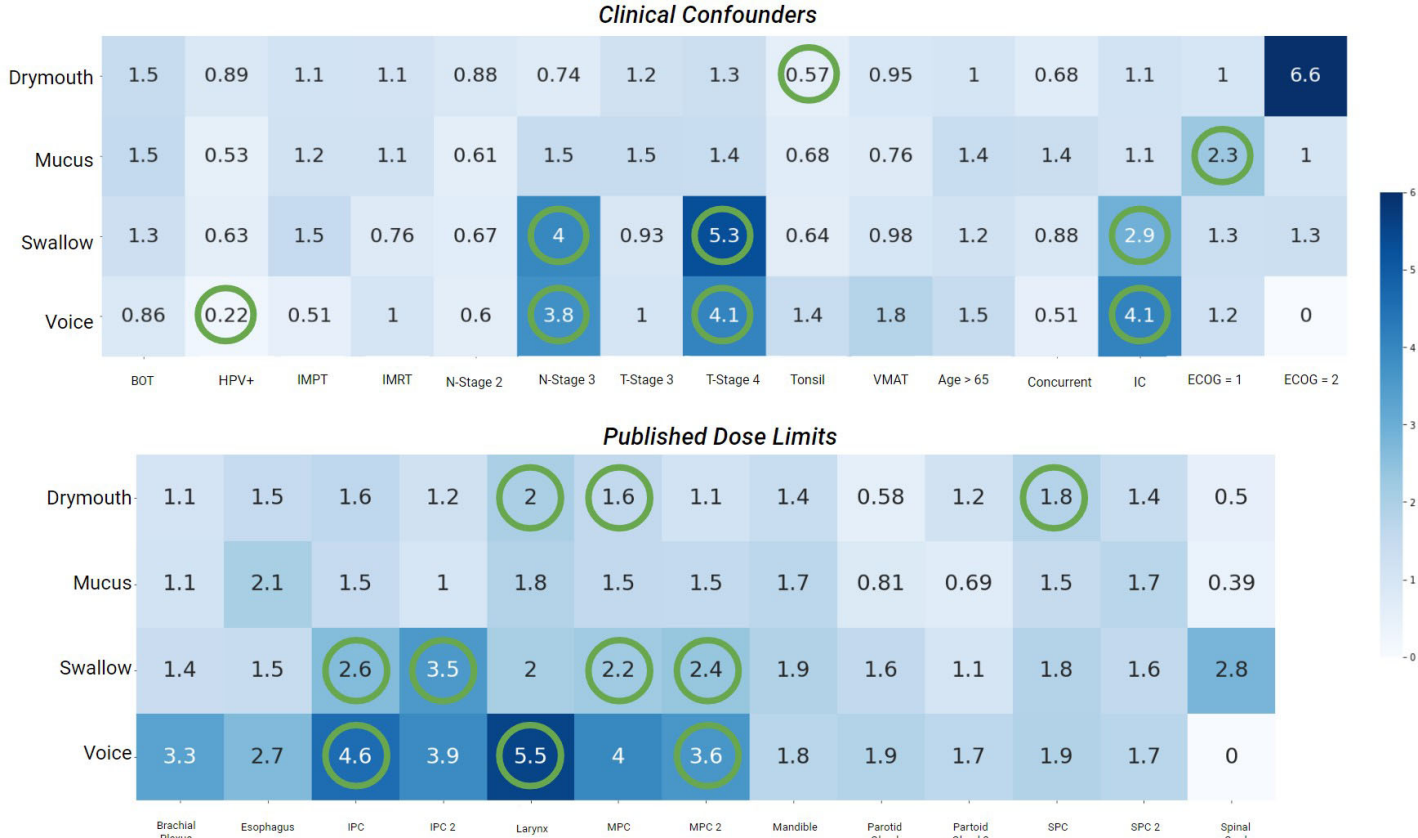
Heatmap of odds-ratios from fishers-exact test between late severe (> 4) ratings for each symptom, and confounders used in the data, (top) as well as published dose limits. Statistically significant values (p <.05) are marked with green circles. Values < 1 indicates lower than average risk while values > 1 indicate above average risk. BOT, Subsite at Base of Tongue; HPV+, HPV/p16 positive; IMPT, Intensity Modulated Proton Therapy); IMRT, Intensity Modulated Radiation Therapy; Tonsil, Subsite at Tonsil; VMAT, Volumetric Modulated Arc Therapy; Concurrent, Chemotherapy concurrent with radiation therapy; IC, Induction Chemotherapy; ECOG, Eastern Cooperative Oncology Group Performance Score.

Results for the correlation tests between baseline confounders, existing dose guidelines, and late severe symptoms are shown in (**Figure 3**). The factors most correlated with severe drymouth were ECOG performance score >= 2, and primary tumor at the base of the tongue (BOT). Oddly, T-stage 4 was negatively correlated with drymouth, while the less-severe T-stage 3 was positively correlated. The strongest predictors of negative outcomes are high doses to the larynx and superior pharyngeal constrictor, which are traditionally associated with swallowing complications and not drymouth. The dose limits to the parotid glands intended to predict xerostomia were negatively correlated with high drymouth, which is likely since most patients whose doses were within acceptable limits were in the low-dose cluster, which had anomalously high rates of drymouth relative to the moderate dose group (38.3% vs 92.92%, respectively).

### 3.2 Cluster analysis

The final parameters for each outcome are shown in (**Table 1**). Interestingly, we found similar simplified rules for predicting late severe voice dysfunction (IPC V55 > 34) and late severe swallowing issues (IPC V50 > 40). Similarly, rules for the high-risk mucus and drymouth clusters show similar rules for thresholds to the contralateral parotid glands (V45 > 61 and V50 > 48), and for the contralateral parotid gland (V45 > 0). Notably, the optimal DVH values were lowest for predicting drymouth than other symptoms with values ranging from V25-V65, compared to V20-V60 for drymouth. Clusters for swallow and voice also had higher optimal DVH values, and generally included more muscles instead of salivary glands.

**Figure 4:**
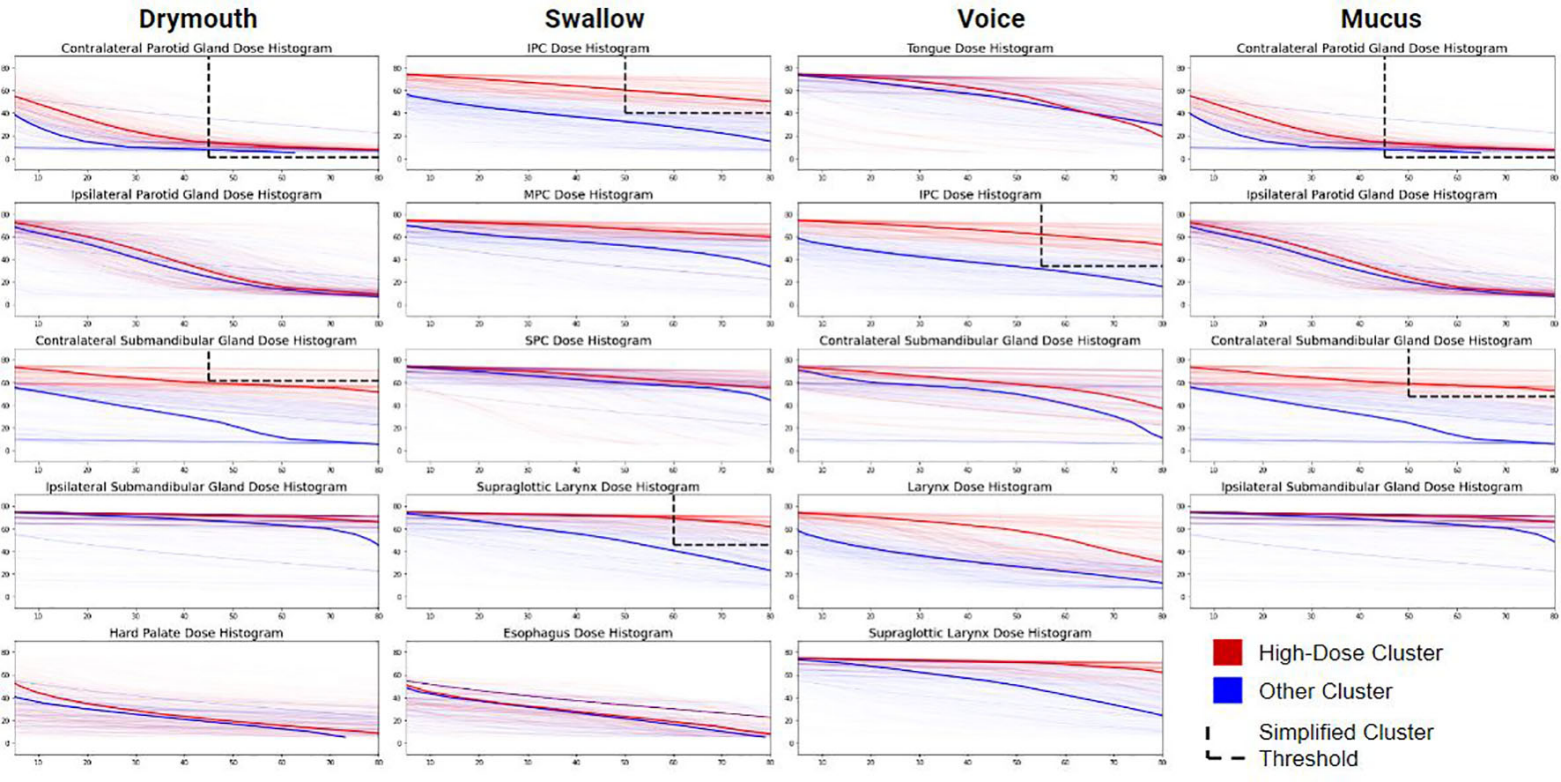
Comparison of Dose-volume features between patients in each high-dose cluster (red) and those in low- or moderate-dose clusters (blue). Each plot shows the dose-volume histogram for each patient. Darker lines show the median values within each group. Dashed lines show the thresholds used for producing the simplified cluster, excluding rules that use max-dose to the ROI. Patient histograms that pass through the upper-right window of all plots in their row are in the simplified high-dose cluster.

Comparison of high-dose and low/moderate-dose-volume histograms of the organs used for the high-dose clusters are in (**Figure 4**). We can see that rules generally correspond to the ROIs that show the highest difference in mean dose between high- and low/moderate-dose groups. We see larger separations for the contralateral submandibular glands, inferior pharyngeal constrictors, and supraglottic larynx. We can also see that in the high-risk group, mean dose to the submandibular glands tends to be relatively high even at 80% penetration, while the dose to the dose to the parotid gland will drop off to low or zero values at around 45% penetration for the low/moderate dose groups. We also see relatively high levels of dose for the MPC and SPC (**Figure 4**-swallow column) even at 80% penetration with limited dropoff.

**Figure 5:**
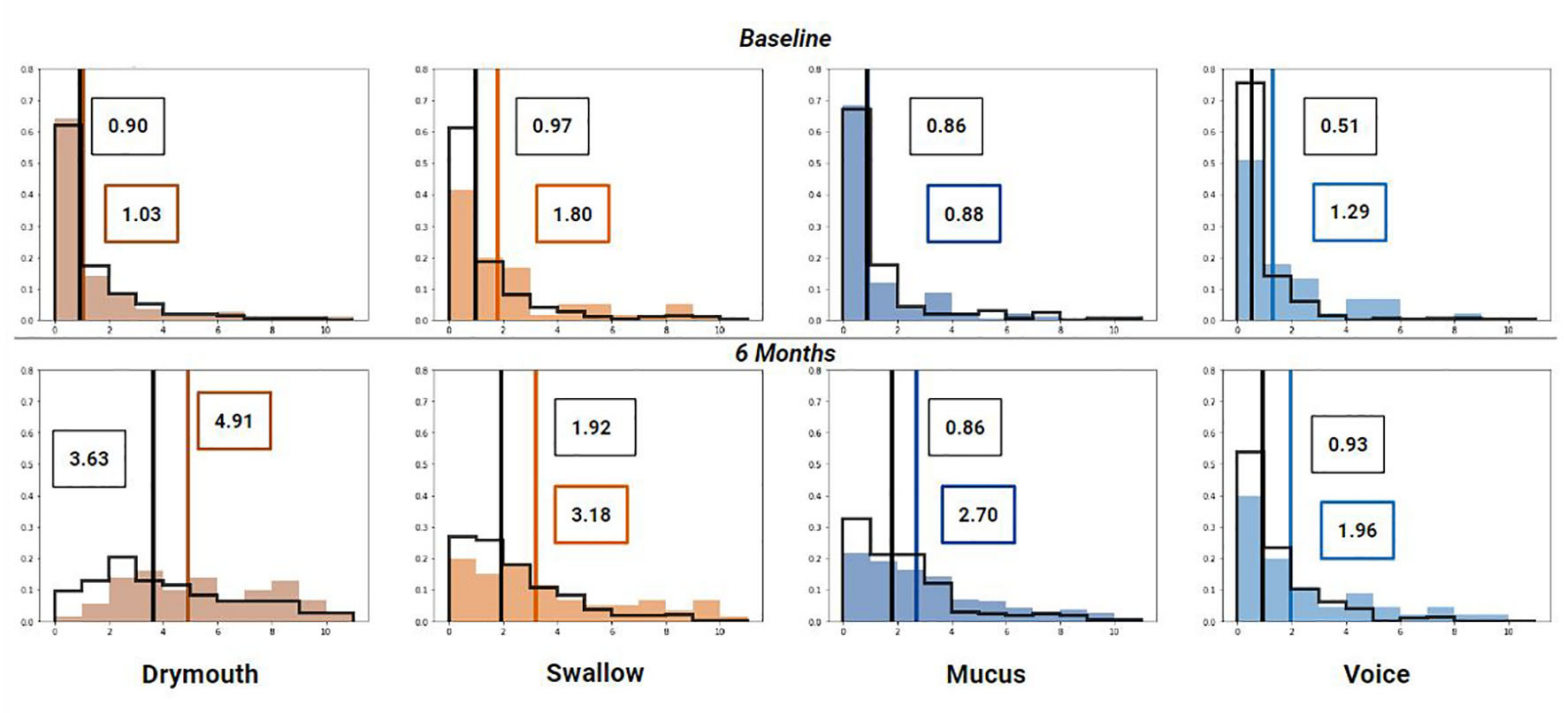
Histogram of symptom ratings before treatment (top), 6 week (middle) and 6 months (bottom) after treatment for each cluster (colored bars) compared to the rest of the cohort (black outline). Lines show median rating for patients within (colored) and patients not in the cluster (black). Mean values for high-dose clusters are labeled in colored boxes while the moderate/low dose clusters are labeled with black boxes.

The distribution of symptoms at the start of RT treatment and at 6 months for each high-risk and low/moderate risk groups are shown in (**Figure 5**). Mean ratings for all groups increase between baseline and 6 months, although the difference in change is higher for the high-dose groups. All high-dose clusters show a slightly higher mean symptom rating at baseline than the low/moderate dose groups, with differences of.14,.83,.01, and.78 for drymouth, swallow, mucus, and voice, respectively. This difference increases at 6 months for all cases to 1.27,.126,.91, and 1.02 for drymouth, swallow, mucus, and voice, respectively. The larger baseline difference for swallow and voice likely corresponds to the higher rates of stages T4 and N3 in these groups at the start of treatment, which we don’t see in drymouth or mucus. The most significant change is in the high-dose drymouth group, which has a mean symptom rating increase of 3.87 between baseline and 6-months.

### 3.3 LRT test results

Results for LRT tests on all outcomes with clinical confounders are reported in (**Table 5**). All outcomes show significant (<.01) correlation between 3-level cluster stratifications and severe late symptoms. When considering the change from baseline rating, we have significant correlations for the high-dose clusters with all outcomes except for “voice”, which may be because we only have 6 patients with a change in voice ratings above 4 in the dataset (1.7%) (**Table 3**).

**Table 5:** Results from LRT tests for severe late drymouth and severe late change in drymouth for swallowing, mucus, and voice outcomes using their clusters.

Outcome		Swallow			Mucus			Voice			Drymouth		
		All	HD	SHD	All	HD	SHD	All	HD	SHD	All	HD	SHD
Rating > 4	P-value	0.001	0.002	0.010	0.003	0.001	0.010	0.004	0.009	0.000	0.000	0.000	0.000
	Odds Ratio	N/A	5.129	3.625	N/A	3.182	2.373	N/A	8.987	19.749	N/A	2.942	2.767
	ΔAIC	-10.6	-7.6	-4.7	-8.0	-9.3	-4.7	-7.2	-4.8	-11.1	-12.3	-14.2	-12.0
	ΔBIC	-2.9	-3.7	-0.9	-0.2	-5.4	-0.8	0.5	-0.9	-7.2	-4.6	-10.4	-8.2
ΔRating > 4	P-value	0.002	0.014	0.028	0.002	0.001	0.032	0.046	0.976	0.409	0.009	0.002	0.002
	Odds Ratio	NA	4.726	3.762	NA	3.382	2.171	NA	0.960	2.559	NA	2.382	2.447
	ΔAIC	-8.8	-4.1	-2.8	-8.1	-8.4	-2.6	-2.1	2.0	1.3	-5.4	-7.3	-7.4
	ΔBIC	-1.1	-0.2	1.0	-0.4	-4.5	1.3	5.6	5.9	5.2	2.3	-3.5	-3.6

All) all clusters, results do not include odds ratio; HD) Highest dose cluster; SHD) Simplified high-dose cluster using the threshold rules; ΔAIC) Change in Aikake Information Criteria from inclusion of the cluster in a regression model; ΔBIC) Change in Bayesian Information Criteria from inclusion of the cluster in a regression model.

For absolute outcomes (rating > 4), Drymouth high-dose (HD) and simplified high-dose (SHD) clusters had the highest significance level (p <.0001) with odds-ratios of 2.942 and 2.767 for severe late drymouth, respectively. Voice had the highest odds-ratios of all symptoms for severe voice dysfunction with values of 8.99 and 19.75 for the HD and SHD, respectively (p <.01). Swallow HD and SHD clusters had odds ratios of 5.129 (p = .002) and 3.625 (p = .01), respectively. Finally, mucus HD and SHD clusters had odds ratios of 3.18 (p = .001) and 2.37 (p = .01), respectively.

For relative outcomes (rating change from baseline > 4), we see similar or slightly lower odds ratios but lower p-values, due to the smaller number of measured outcomes, for Drymouth HD (OR = 2.38, p = .002), Drymouth SHD (OR = 2.447, p <.002), Swallow HD (OR = 4.73, p = .014), Swallow SHD (OR = 3.76, p = .028), Mucus HD (OR = 3.382, p <.001), and Mucus SHD (p = 2.17, p = .032). However, there is no correlation between Voice HD (OR = .96, p = .96) or Voice SHD (OR = 2.55, p = .42) and change in voice ratings > 4.

Comparing 3-level cluster stratifications, HD cluster, and SHD clusters, HD clusters tend to perform slightly better, except in the case of predicting severe late drymouth and severe late voice, in which the SHD clusters do marginally better. Inclusion of the 3-level stratifications over the High-dose only clusters didn’t have a notable difference in significance level. Except for change in swallow >4 from baseline, 3-level stratification tended to perform worse in terms of change in Bayesian Information Criteria, suggesting that majority of the information gain comes from the high-dose clusters.

### 3.4 Cross-validation results

**Figure 6:**
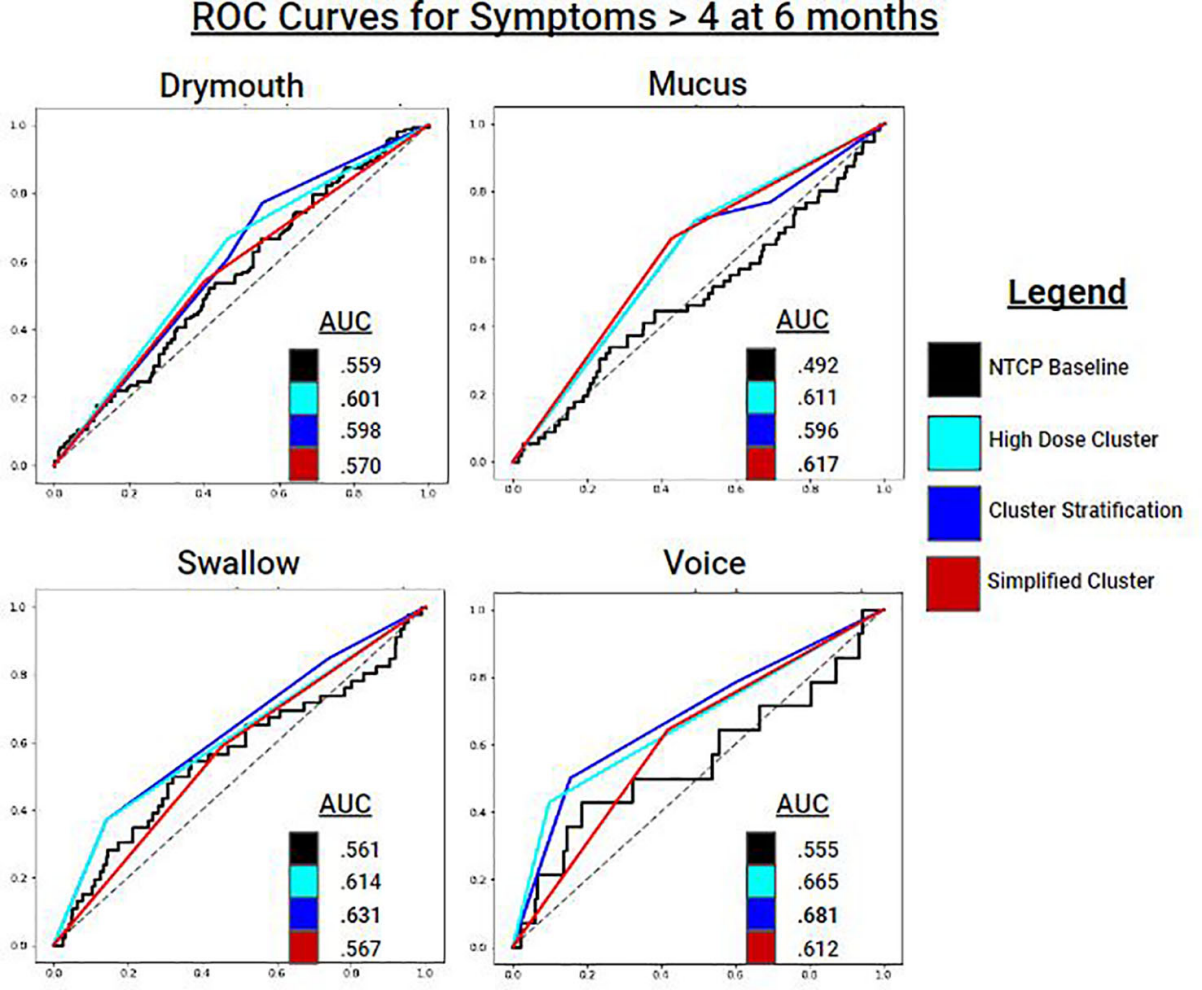
ROC Curves for predicting symptom ratings > 4 at 6 months for each symptom ratings. Cluster stratifications include: all clusters (blue), the high dose cluster (cyan), and the simplified high dose cluster (red). Baseline models for comparison are NTCP logistic regression models which includes dosimetric variables and clinical variables.

We report results from performing cross-validation for several alternative patient outcomes in (**Table 6**). ROC curves for each outcome on severe ratings are shown in (**Figure 6**). In terms of ROC and MCC, cluster stratification (3 clusters) outperformed baseline NTCP models for all outcomes. Performance differences between only the high-dose clusters (HD), simplified clusters (SHD), and all clusters (3-level stratification) were mixed, with the high-dose cluster outperforming all clusters for late mucus and drymouth, but not voice or swallow.

**Table 6:** Area-under the curve score (AUC) and Mathew’s correlation coefficient (MCC) scores from 5-fold cross-validation testing using cluster stratification and NTCP models for severe (> 4) self-reported symptoms at 6 months.

Outcome		Swallow				Mucus				Voice				Drymouth			
Metric		All	NTCP	HD	SHD	All	NTCP	HD	SHD	All	NTCP	HD	SHD	All	NTCP	HD	SHD
Rating > 4	AUC	0.63	0.56	0.61	0.57	0.60	0.49	0.61	0.62	0.68	0.56	0.67	0.61	0.60	0.56	0.60	0.57
	MCC	0.20	-0.03	0.20	0.09	0.16	-0.04	0.16	0.17	0.18	-0.02	0.21	0.09	0.14	0.06	0.20	0.14
ΔRating > 4	AUC	0.61	0.50	0.60	0.57	0.63	0.50	0.64	0.64	0.52	0.52	0.53	0.45	0.58	0.57	0.58	0.55
	MCC	0.14	-0.02	0.14	0.08	0.19	-0.03	0.19	0.18	0.00	-0.01	0.02	-0.03	0.11	0.08	0.16	0.09

All) Stratification with all clusters; NTCP) Fitted NTCP logistic regression model; HD) Stratification with only the high-dose cluster; SHD) Stratification with only the simplified high-dose cluster rules.

For Drymouth outcomes, the HD cluster alone performed the best for all measures, with an AUC of.6 for severe drymouth vs.56 for the NTCP + clinical covariates model. Using the 3-level stratification achieved the same AUC score as the HD cluster, but lower MCC, due to the higher number of clusters. SHD slightly outperformed the NTCP model for absolute rating > 4 (AUC.57 vs.56), but not for change in rating > 4 (AUC.55 vs.57), although the SHD had a higher MCC for both outcomes.

For Swallow, 3-level stratifications performed the best in terms of AUC for rating > 4 (AUC = .63) and change in rating > 4 (AUC = .61). In all cases for swallow, the 3-clusters performed the best, followed by the HD, SHD, and NTCP models performed the worst.

For Mucus, the SHD performed the best in terms of AUC for both Mucus > 4 (AUC = .62) and change from baseline > 4 (AUC = .64). Voice had mixed results in terms of performance. For Voice > 4, the 3-level model performed the best (AUC = .68), followed by HD (AUC = .67), and SHD (AUC = .61), and finally the NTCP model (AUC = .56). For change from baseline, all models performed close to chance due to the lower number of positives, with the highest performance from HD (AUC = .53).

## 4 Discussion

Our results demonstrate the benefits of grouping OPC RT patients based on multi-organ key 3D dose spatial distribution metrics related to patient outcomes. By identifying organs that may serve as failure points for essential functions, we were able to identify a high-dose, high-risk group of patients. Both the original high-dose cluster and the simplified version of this cluster are strongly correlated with the severity of self-reported symptoms that persist up to 6 months after treatment and improve predictive models after accounting for clinical confounders and overfitting. This methodology can serve as a valuable tool for identifying potential causes of lasting toxicities because of radiation-induced damage that outperforms existing models and can be used alongside NTCP risk prediction models. Additionally, we provide a rule mining algorithm that can simplify our rule set into a set of actionable dose thresholds that can be used without access to the original model.

Existing approaches for normal tissue toxicity probability (NTCP) calculation for risk prediction rely on summary dosimetric parameters (11), such as generalized equivalent uniform dose (32), maximum, or mean dose to a region of interest. Normal Tissue Complication Probability (NTCP) models can address three-dimensional dose distributions to individual organs to predict outcomes. Existing models suffer from limitations imposed by challenges of dealing with correlated dose features, assumptions of linear relationships between dose and effect, and reliance on simplifying 3-dimensional dose distributions to a single unit (33). We attempt to address these issues with the use of clustering on 2-dimensional dose-volume histograms, which allows us to capture patterns in the dose distribution that encompass relationships between many correlated features in a way that does not assume linearity or uncorrelated dose features. Additionally, our simplified stratifications are transparent, which makes them more convenient to use when incorporating them into existing treatment guidelines and accounting for patient-specific information. Finally, we note that while we directly compare our model to NTCP models, these metrics can be used alongside each other, as NTCP models are designed for use in calculating specific risks when using dose planning software, while our methods are designed to provide convenient risk stratification for identifying high-risk patients and giving simple dosing guidelines.

Outside of NTCP models, the most common risk stratification for OPC patients is AJCC TNM staging. T, N, and M-staging criteria consider the size and spread of the primary tumors, secondary tumors, and distant metastasis, respectively, to predict survival (19). While TNM staging is not directly related to late toxicity risk, it can serve as a proxy for the aggressiveness of treatment and is correlated with radiation-associated dysphagia in patient outcomes (28).

In our cohort, the predominant lasting toxicity was severe drymouth, which occurred in 43.8% of patients, while only 5.2% of patients reported no drymouth at 6 months, which makes it of particular interest for clinical applications. Our cluster parameters for drymouth include the submandibular glands, and the hard palate, which are all possibly causally linked to patients experiencing drymouth. When considering the simplified cluster, we found using the V45 to the contralateral submandibular gland and the V45 to the contralateral parotid gland achieved a sensitivity and specificity of.89 and.98, respectively. This suggests that treatment planning should prioritize reducing the dose delivered bilaterally to the submandibular salivary glands, as well as sparing at least 55% of the contralateral salivary gland from irradiation. These findings suggest that damage to both sets of salivary glands, rather than one, is a major factor in determining severe drymouth, as sparing a single set of glands may be able to mitigate the severity of experienced drymouth. At the same time, high dose to the contralateral side of the head is also correlated with larger and more extensive tumor spread, which may be a confounding factor that we would like to investigate in future work (28).

When comparing our clusters for different symptoms we see that the optimal parameters for predicting both drymouth and mucus include the parotid glands and submandibular glands, which indicate that mucosal dysfunction may be related to drymouth. Our parameters for swallow and voice issues consider larger sets of muscles closer to the area around the neck and base of the tongue, while mucus and drymouth focus on salivary glands in the mouth. Additionally, we see that the optimal parameters for swallow and voice consider radiation at larger levels of penetration into the volume (V30-V65) and contain smaller high-risk clusters (**Table 6**). This may reflect a greater tolerance in muscle tissue over salivary glands to radiation. Overall, the alternative symptoms considered were reported as severe (> 5) less frequently than drymouth, which may explain the larger p-values on LRT tests relative to drymouth, even when performed on predictive models was good for high-dose and simplified high-dose clusters.

**Figure 7:**
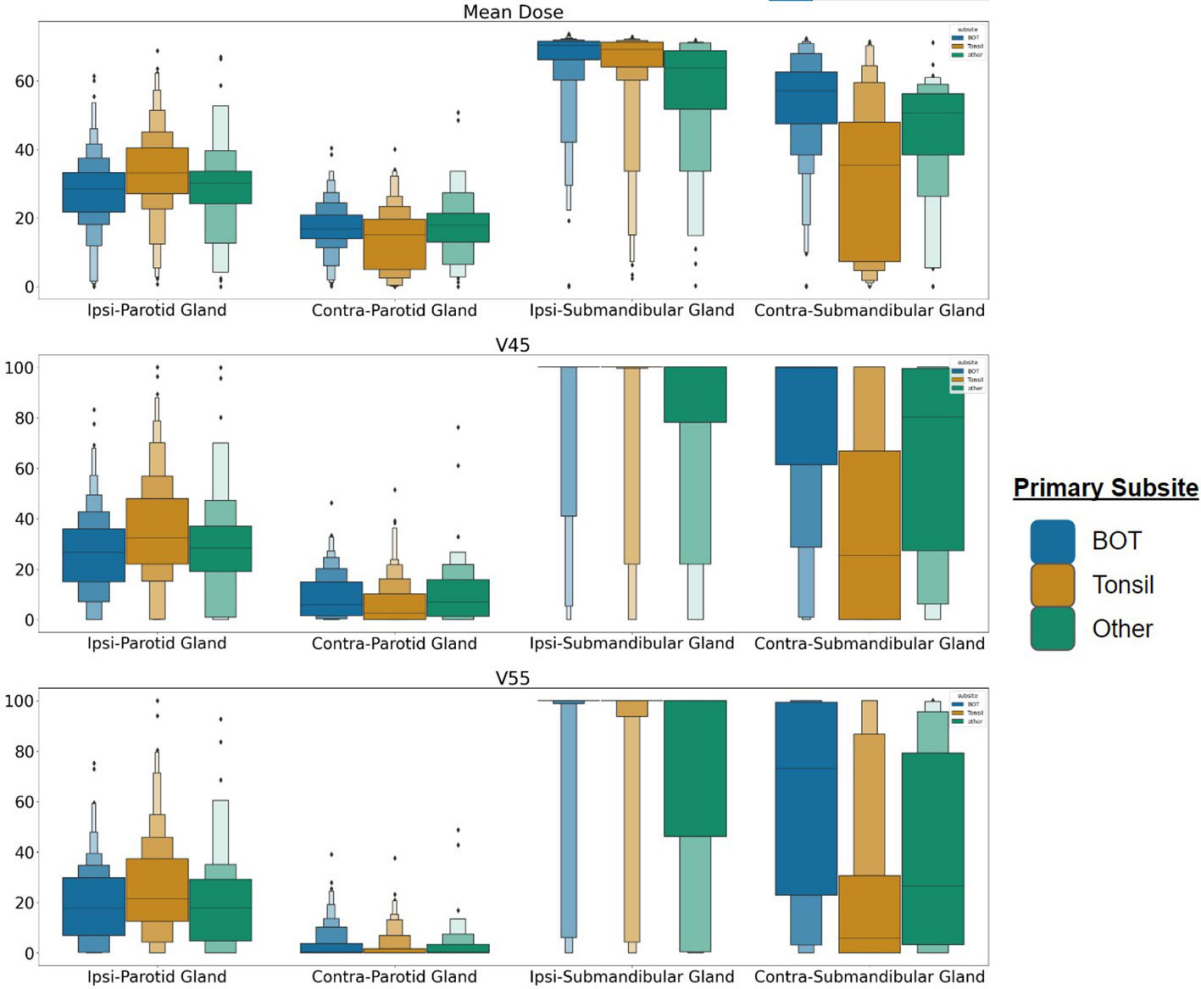
Dose distribution for patients with tumors at the BOT, Tonsil, and any other subsite for the parotid and submandibular salivary glands. Each row represents Mean Dose, V45, and V55, respectively. BOT subsite is associated with higher average doses to the contralateral submandibular glands, suggesting more frequent bilateral irradiation. Each rectangle in the plot represents the value range for a quantile.

While our models represent an improvement over existing tools, overall performance remains relatively low, with clinical baseline models performing only slightly above chance, which may reflect the difficulty in precisely identifying patients at high risk of symptoms using only EHR and dosimetric data. Notably, the previously suggested dose limits for the parotid gland to limit xerostomia are not correlated with drymouth, with most outcomes yielding a negative odds ratio, likely due to other confounders in the data. Of our confounders, we found that the strongest predictors were ECOG performance score, having a tumor at the base-of-tongue, and receiving proton therapy. The relationship between tumors at the BOT supports the theory that higher doses to the submandibular glands are related to drymouth. Preliminary analysis suggests that patients with a primary subsite at the BOT are associated with higher doses to the contralateral submandibular gland (**Figure 7**), with an average mean dose of 66Gy and 54Gy to the ipsilateral and contralateral submandibular glands, respectively, vs 62Gy and 34Gy for other subsites. On the other hand, BOT tumors are not associated with higher doses to the parotid glands.

Interestingly, we also found that late T-staging (T4) and N-staging (N3) was strongly predictive of severe swallow and voice dysfunction, but not mucus or drymouth. Both swallow and voice also had a higher difference in baseline symptom ratings between the high-dose and moderate-dose groups, as well as higher rates of tumors at the base-of-tongue. This suggests that there may be additional effects caused by the tumor itself in addition to radiation damage. Regarding treatment modality, we didn’t find a correlation between method and outcomes (**Table 3**), but we did find a correlation between treatment method and cluster, with the HD clusters being a higher portion of patients that received VMAT or IMPT, especially for the swallow and voice clusters.

Our results consider both overall severity at 6 months (rating > 4), as well as severe change in rating relative to baseline ratings (change > 4). The inclusion of the severe change outcome is designed to filter out patients with high baseline symptoms, whose toxicity may not be related to radiation-induced damage. Results show that our model still improves over the baseline in these cases, with a slight decrease in measured effect size, which is likely due to the smaller number of outcomes. However, we don’t find a significant correlation when considering severe change in voice outcomes, which may be because only 1.7% (6) of patients in the data report this outcome (**Table 3**). Additionally, we see that the high-risk clusters have a lower incidence of patients with prior surgery than the main cohort, or the low-risk group. These findings support the idea that the differences in patient outcomes are likely related to radiation-driven effects, and not confounders due to the impact of prior treatment.

With respect to our study’s limitations, while our methodology attempts to identify the organs most likely to have a causal effect on outcomes, the nature of radiation dosing makes identifying causal relationships difficult due to the highly correlated nature of the doses. Spatially adjacent organs have highly correlated doses which makes disentangling their effects difficult without very large datasets. Additionally, our results are sensitive to the choice of dose parameters and require parameter tuning in order to translate our results to other cohorts. Although we focus on HNC cancer here, our method could be generalized to other types of cancer that are linked to radiation-associated side effects, although other localized considerations may need to be taken, such as greater shape variability in the case of bladder cancer. Since the thresholds may be affected both by the specific organ and treatment methods, generalizing these results to other cohorts requires calibration of dose-volume parameters used in the clustering. Additionally, our reliance on imputation for 17% of the baseline symptoms may introduce some bias. Finally, while we attempt to use baseline features to correct for high initial symptoms, this approach may under-count patients whose initial symptoms were caused by the tumor itself as the initial symptoms not due to radiation damage would decrease after completion of treatment.

Future work could also consider modifying the dose distributions on a per-organ basis, as the submandibular glands may have lower threshold tolerances than larger muscles such as the tongue. The model may be further improved by using segmentation of specific sublingual and salivary glands in the mouth, beyond the two sets that we consider. Additionally, while we only consider dose plans prepared before treatment, future research could consider the impact of anatomical data as well as the impact of changes in dose due to temporal anatomical changes in response to treatment (34). Finally, we plan on incorporating additional information that may provide additional insight into patient risks, such as tumor location and bilaterality. Finally, other work may investigate correlating doses to more complicated patterns of symptom progression rather than simply considering late severe symptoms, such as those being investigated in other works such as (35).

In conclusion, our paper presents an unsupervised methodology for identifying patients with high doses to a set of organs, which we have shown are associated with a higher risk of lasting severe symptoms. Our model uses unsupervised Gaussian Mixture Models and approaches based in rule mining to find stratification rules that consider failure points at multiple organs in order to identify high-risk patients.

## Data availability statement

The datasets presented in this study can be found in online repositories. The names of the repository/repositories and accession number(s) can be found below: https://figshare.com/s/323b80aac562a0c910b3.

## Author contributions

All listed co-authors performed the following: 1. Substantial contributions to the conception or design of the work; or the acquisition, analysis, or interpretation of data for the work; 2. Drafting the work or revising it critically for important intellectual content; 3. Final approval of the version to be published; 4. Agreement to be accountable for all aspects of the work in ensuring that questions related to the accuracy or integrity of any part of the work are appropriately investigated and resolved. Specific additional individual cooperative effort contributions to study/manuscript design/execution/interpretation, in addition to all criteria above are listed as follows: AW - performed data processing, designed the clustering and rule ming method, created associated visualizations, and performed statistical analysis and interpretation. LD, MN, AM - direct patient care provision, direct clinical data collection; interpretation and analytic support. GC - supervised statistical analysis, data extraction, graphic construction. MN, CF - analytic support, guarantor of statistical quality. AW, GC, MN, GM - manuscript writing. GC, CF, GM - primary investigator(s); conceived, coordinated, and directed all study activities, responsible for data collection, project integrity, manuscript content and editorial oversight and correspondence
